# Supplementary material for: Baseline Characterization of the Gut Microbiota of Field and Colony Populations of Phlebotomus tobbi and Preliminary Assessment of the Anti-Leishmanial Activity of Cultivable Bacteria
Source: Pathogens. 2026 Jun 23;15(7):658. doi: 10.3390/pathogens15070658 (PMC13416292; doi:10.3390/pathogens15070658)
Supplement: Supplementary file 1 [file pathogens-15-00658-s001.zip › pathogens-4357168-File S1/Taxa2ASV_Decomposer/Family/f__Sphingomonadaceae/abun.plot.top12.pdf]

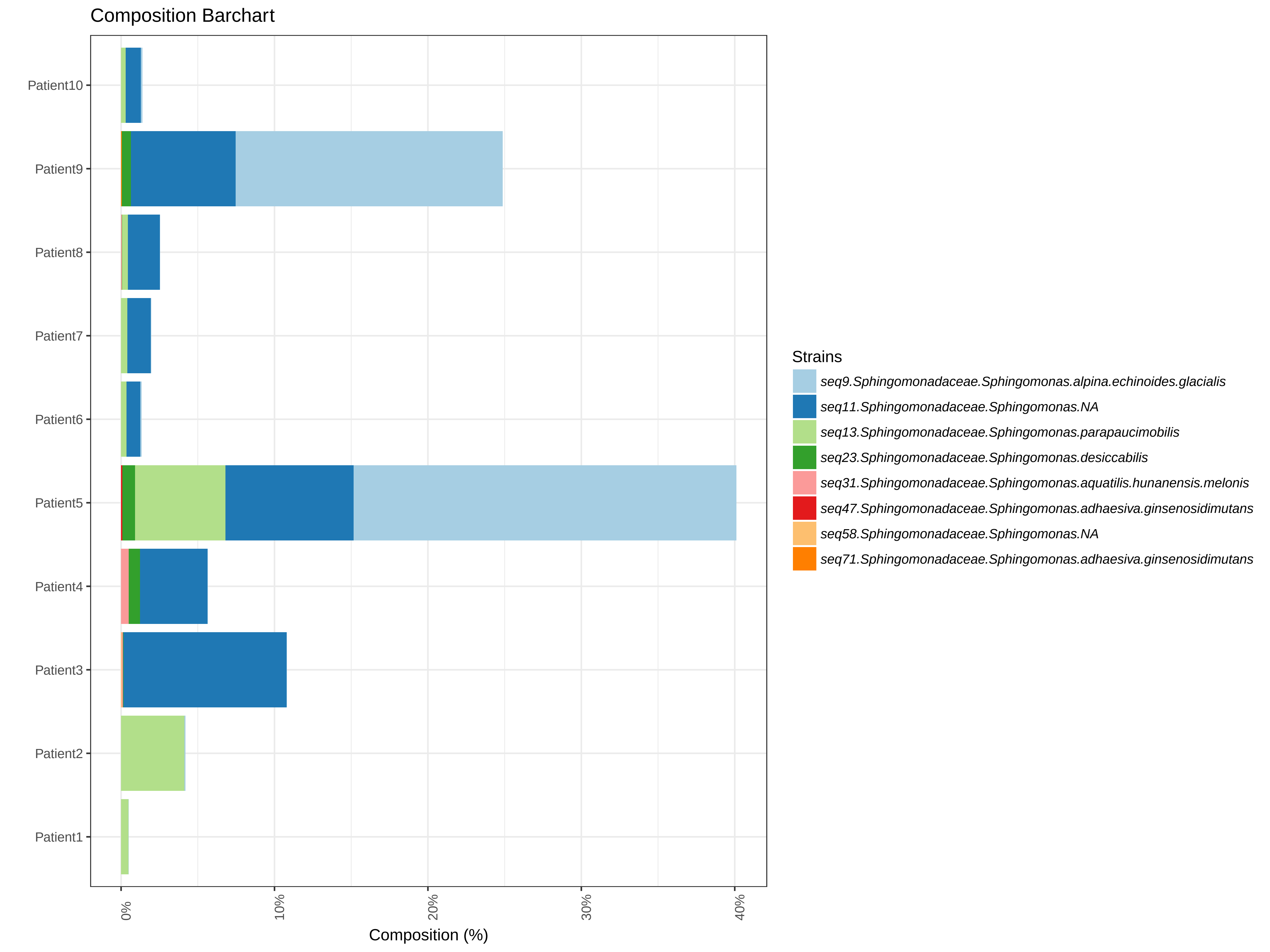

Composition Barchart
Patient10
Patient9
Patient8
Patient7
Patient6
Patient5
Patient4
Patient3
Patient2
Patient1
0%
10%
20%
30%
40%
Composition (%)
Strains
seq9.Sphingomonadaceae.Sphingomonas.alpina.echinoides.glacialis
seq11.Sphingomonadaceae.Sphingomonas.NA
seq13.Sphingomonadaceae.Sphingomonas.parapaucimobilis
seq23.Sphingomonadaceae.Sphingomonas.desiccabilis
seq31.Sphingomonadaceae.Sphingomonas.aquatilis.hunanensis.melonis
seq47.Sphingomonadaceae.Sphingomonas.adhaesiva.ginsenosidimutans
seq58.Sphingomonadaceae.Sphingomonas.NA
seq71.Sphingomonadaceae.Sphingomonas.adhaesiva.ginsenosidimutans
